# Effects of Dietary Supplementation with *Eucommia ulmoides* Extract on Growth Performance, Immune Parameters, and Intestinal Microbiota of Largemouth Bass (*Micropterus salmoides*)

**DOI:** 10.3390/microorganisms14081740

**Published:** 2026-08-07

**Authors:** Ruixian Huo, Hannan Gong, Lifang Cao, Dekun Tang, Yifang Chen, Min Yang, Shina Wei

**Affiliations:** 1 College of Marine Sciences, South China Agricultural University, Guangzhou 510642, China; hrx1582002@163.com (R.H.); 17689735472@163.com (H.G.); 18948120826@163.com (L.C.); 17266230189@163.com (D.T.); 2 Guangdong Kingkey Smart Agri Technology Co., Ltd., Dongguan 523000, China; 15916889838@163.com; 3 Nansha-South China Agricultural University Fishery Research Institute, Guangzhou 511457, China

**Keywords:** *Eucommia ulmoides* extract, largemouth bass virus, gut microbiota, antioxidant response, immune response

## Abstract

The largemouth bass (*Micropterus salmoides*) is an important freshwater aquaculture species in China, but the increasing incidence of disease under intensive production conditions has become a major constraint on its sustainable development. This study evaluated the effects of dietary *Eucommia ulmoides* extract (ELE) supplementation on growth performance, immune and antioxidant responses, intestinal health, gut microbiota, and resistance to largemouth bass virus (LMBV) in juvenile largemouth bass. During a 56-day feeding trial, fish with an initial body weight of 13.14 ± 0.11 g were fed diets containing 0, 30, 60, 90, 120, or 150 mg ELE/kg, with three replicate nets per treatment. Dietary supplementation with 60–90 mg/kg ELE significantly increased weight gain rate (*p* < 0.05). ELE also improved selected serum antioxidant and immune indices, with the most pronounced responses generally observed at intermediate supplementation levels. In the intestine, the increased Nrf2 and decreased Keap1 mRNA expression were consistent with the potential involvement of Keap1/Nrf2-related redox regulation. ELE also modulated the expression of inflammation- and apoptosis-related genes and increased the expression of selected tight-junction-related genes, suggesting a potential contribution to intestinal inflammatory regulation and barrier maintenance. In addition, ELE supplementation was associated with shifts in bacterial diversity and community composition, although the responses varied among supplementation levels. Following LMBV challenge, the 90 mg/kg group showed the highest observed protection, accompanied by lower splenic expression of the largemouth bass virus major capsid protein (LMBV-MCP) and proinflammatory gene expression, suggesting reduced viral activity and host inflammatory responses. Overall, dietary supplementation with 60–90 mg/kg ELE elicited the most favorable combined responses and may contribute to the regulation of growth, antioxidant and immune status, and intestinal homeostasis in largemouth bass.

## 1. Introduction

Largemouth bass (*Micropterus salmoides*) is a commercially important species, owing to its appealing taste, high market value, and lack of intermuscular bones [1]. In recent years, its aquaculture industry has grown rapidly and has emerged as a key component of the aquaculture sector [2]. However, the continuous expansion of scale and the widespread adoption of intensive, high-density farming have led to environmental degradation and increased stocking pressures. This has compromised fish disease resistance and resulted in frequent disease outbreaks [3].

Among the infectious diseases affecting this species, largemouth bass virus (LMBV), a number of the genus *Ranavirus* within the family *lridoviridae*, has become an important threat to largemouth bass aquaculture [4]. Affected fish may exhibit skin ulceration, muscle necrosis, hemorrhage, splenomegaly, and hepatic lesions, and severe infection can result in substantial mortality and economic losses [4,5]. The severity of LMBV infection is influenced by environmental conditions, particularly water temperature: experimental infection with the LMBV FJ_22109 strain resulted in 100% cumulative mortality within 7 days at 29 °C [5]. Current prevention primarily relies on biosecurity and general health management. Although several injectable, oral, immersion, and DNA vaccine strategies have been investigated, most remain under experimental evaluation, and their practical application in large-scale aquaculture remains limited [4,6]. Moreover, specific antiviral interventions suitable for routine aquaculture use remain limited. These constraints highlight the need to develop safe and practical nutritional strategies that may improve host resistance and reduce the adverse effects associated with LMBV infection.

*Eucommia ulmoides* is a traditional Chinese medicinal herb, and its leaf extract contains abundant polyphenolic constituents with potential biological activities [7]. Evidence from aquatic species suggests that ELE may influence growth, antioxidant status, immune-related responses, and intestinal health. In large yellow croaker (*Larimichthys crocea*) larvae, dietary supplementation with 1.0% ELE improved growth performance and selected antioxidant and innate immune indices, whereas supplementation with 0.5–1.0% ELE decreased the transcript levels of *cox-2*, *il-1β*, and *il-6* [8]. In channel catfish (*lctalurus punctatus*) fed a high-fat diet, supplementation with 0.1–0.2% ELE affected hepatic lipid metabolism and inflammatory responses and was associated with enhanced intestinal antioxidant capacity, altered tight-junction-related gene expression and gut microbial composition, and increased resistance to *Aeromonas hydrophila* infection [9]. More recently, dietary ELE was reported to affect growth, liver and intestinal health indicators, gut microbiota composition, and resistance to *A. hydrophila* in juvenile largemouth bass [10]. Regarding its possible biological basis, dietary ELE increased selected antioxidant enzyme activities and altered the mRNA and protein expression of Nrf2 and TNF-α in piglets, suggesting possible involvement in Nrf2-related redox regulation and TNF-α-associated inflammatory responses [11]. Chlorogenic acid, an important polyphenolic constituent of *E. ulmoides*, has also been investigated directly in aquatic animals. In largemouth bass fed high-fat diets, dietary chlorogenic acid improved antioxidant-related indices and attenuated hepatic inflammatory responses [12]. In common carp (*Cyprinus carpio*), chlorogenic acid increased antioxidant enzyme activities, upregulated the expression of Nrf2 and HO-1, and decreased the transcript levels of NF-κB, TNF-α, and IL-1β, suggesting its involvement in redox and inflammatory regulation [13]. Consistent with these anti-inflammatory effects, chlorogenic acid attenuated LPS-induced increases in IL-6, IL-8, TNF-α, and IL-1β expression in bovine mammary epithelial cells, an effect associated with NF-κB regulation [14]. Furthermore, in human rhabdomyosarcoma (RD) cells, chlorogenic acid inhibited enterovirus 71 replication during the early post-entry stage and reduced the secretion of IL-6, TNF-α, IFN-γ, and MCP-1 [15]. Collectively, these findings provide an aquaculture-specific and biological basis for evaluating ELE; however, whether dietary ELE affects resistance to LMBV infection and the associated antioxidant, inflammatory, intestinal, and microbial responses in largemouth bass remains unclear.

Therefore, this study investigated the effects of dietary ELE supplementation on the growth performance, immune parameters, and intestinal health of largemouth bass, as well as its protective effect against LMBV infection, to explore its potential as an aquatic feed additive.

## 2. Materials and Methods

### 2.1. Experimental Diets

Six experimental diets were prepared with white fishmeal and soybean meal as protein sources and fish oil and soybean oil as lipid sources, supplemented with 0 mg/kg, 30 mg/kg, 60 mg/kg, 90 mg/kg, 120 mg/kg and 150 mg/kg *Eucommia ulmoides* extract (ELE), designated ele0, ele30, ele60, ele90, ele120, ele150, respectively. These inclusion levels refer to the mass of the ELE product rather than the chlorogenic acid-equivalent dose. The ELE was purchased from Xian Orchid Biotechnology Co., Ltd. (Xi’an, China) and was standardized to contain 10% chlorogenic acid (*w*/*w*) as determined by HPLC (manufacturer’s certificate of analysis). All the ingredients were mechanically pulverized by passing through a 60-mesh sieve (the proportion of ultrafine particles was less than 5%), followed by precise weighing and uniform mixing using a Hobart-type mixer (model SZ250, Guangzhou AsahiZhong Food Co., Ltd., Guangzhou, China). After mixing, fish oil, soybean oil, and soy lecithin were added to the mixture, stirred well, and then pure water was added to make the dough. The dough was extruded into a feed with a diameter of 2.0 mm using a twin-screw extruder (model F-26, South China University of Technology, Guangzhou, China). The extruder was operated without external heating, and the ambient temperature during extrusion was approximately 34 °C. Finally, the feeds were air-dried at room temperature and stored in a refrigerator at −20 °C until use. The formulation and composition of the test feeds are shown in Table 1.

### 2.2. Experimental Animals and Feeding Trial

Juvenile largemouth bass were supplied by Guangdong Kandar Marine Farm Research Institute (Dongguan, China). After fasting for 24 h, 540 healthy and disease-free largemouth bass with an average weight of 13.14 ± 0.11 g (mean ± SD) were randomly assigned to 6 treatment groups (0, 30, 60, 90, 120, 150 mg/kg ELE), each with 3 replicate nets, giving a total of 18 nets with 30 fish per net. All nets were of the same size (1.2 m × 0.8 m × 1.0 m), giving a volume of 0.96 m^3^ per net. The stocking density was 31.3 fish/m^3^. All nets were placed in the same test pond. An 8-week feeding trial was conducted with twice-daily feeding at 6:30 and 16:30. Every two weeks, all fish in each net were batch-weighed, and the daily feeding rate was set at 3% of the most recently measured combined body weight of the fish in that net. Feed consumption and fish mortality were recorded per net.

### 2.3. Sample Collection

At the end of the 8-week feeding trial, the fish were fasted for 24 h and then anesthetized with 100 mg/L MS-222 (Sigma, St. Louis, MO, USA). All fish in each net were counted and batch-weighed. The mean final body weight for each net was calculated by dividing the combined body weight of the surviving fish by the number of surviving fish. These net-level data were used to calculate the weight gain rate, specific growth rate, and feed conversion ratio, with each net regarded as the experimental unit. In addition, three fish were randomly selected from each net for individual body weight and body length measurements and were subsequently used for the determination of morphometric indices and tissue sampling. These individual measurements were not used to estimate net-level growth performance.

Six fish were randomly selected from each replicate net for blood collection, resulting in a total of 18 fish per dietary treatment. Blood was collected from the caudal vasculature using a syringe coated with 1% sodium heparin (the syringe was rinsed and then emptied, leaving only a thin coating). The collected blood was transferred into a centrifuge tube and allowed to rest at a constant temperature of 4 °C for 12 h, then centrifuged at 1000× *g* for 10 min. The plasma was collected and stored in an ultralow-temperature freezer at −80 °C for subsequent analysis of plasma biochemical indices.

Six test fish were randomly selected from each replicate group. After blood collection, the fish were dissected on ice, and the entire intestine (from the pyloric caeca to the anus) was removed, weighed, placed into cryotubes, and stored at −20 °C for determination of intestinal antioxidant enzyme activities. Finally, three additional fish were randomly selected from each replicate net (nine fish per dietary treatment). The intestinal contents were aseptically collected, and the contents from the three fish within the same net were pooled into one sterile tube to generate a composite sample. Thus, three independent composite samples were obtained per dietary treatment, corresponding to the three replicate nets. This pooling strategy was used to obtain a representative net-level microbial profile and to maintain consistency with the replicate net as the experimental unit. The pooled intestinal-content samples were immediately frozen in liquid nitrogen and stored at −80 °C without RNA stabilization solution until microbial DNA extraction. In contrast, intestinal tissue samples collected for gene-expression analysis were immersed in RNA stabilization solution and stored at −80 °C until RNA extraction.

All sampling procedures were completed within approximately 4 h, from 14:00 to 18:00 on the same day. To minimize potential effects of sampling time, replicate nets from the different dietary treatments were sampled in randomized order. Sampling was conducted by the same trained team using standardized procedures, with fixed personnel assigned to anesthesia and measurement, blood collection, dissection, and sample preservation. All samples for each biochemical or gene-expression assay were analyzed by the same operator under identical experimental conditions.

### 2.4. Calculation of Growth Indicators

The growth rate, specific growth rate, feed conversion ratio, and survival rate of the experimental fish were calculated as follows.

(1) Weight gain rate (WGR, %) = 100 × [(final mean weight − initial mean weight)/initial mean weight].
(2) Specific growth rate (SGR, %) = 100 × [(ln final mean weight − ln initial mean weight)/56 days].
(3) Feed conversion ratio (FCR) = dry feed intake (g)/body weight gain (g).
(4) Survival (%) = (final number of fish/initial number of fish) × 100.

### 2.5. Hematological Parameter Analysis

Plasma biochemical and immune parameters, including alanine aminotransferase (ALT), aspartate aminotransferase (AST), lipopolysaccharide (LPS), endothelin 1 (ET-1), high-density-lipoprotein (HDL) cholesterol, low-density-lipoprotein (LDL) cholesterol, total cholesterol (TC), triglycerides (TGs), total protein (TP), complement 3 (C3), complement 4 (C4), immunoglobulin M (IgM), peroxidase enzyme (POD), and acid phosphatase (ACP). The kits were purchased from Beijing Huaying Institute of Biotechnology (Beijing, China) and were determined by colorimetric assay on an enzyme labeling apparatus (Wuxi Huawei Delang Instrument Co., Ltd., Wuxi, China).

### 2.6. Intestinal Antioxidant Enzyme Activity Analysis

Superoxide dismutase (SOD), catalase (CAT), total antioxidant capacity (T-AOC), reduced glutathione (GSH), malondialdehyde (MAD), and glutathione peroxidase (GSH-Px) were determined using analytical kits purchased from Built Biological Engineering Research Institute (Nanjing, China). The kit for tissue protein concentration was purchased from Biyuntian Biotechnology (Shanghai, China). The colorimetric assay was performed on an enzyme marker (Varioskan LUX, Thermo Fisher Scientific, Shanghai, China) following the instructions.

### 2.7. Intestinal Histology

The hindgut was dehydrated using alcohol of varying concentrations, washed with toluene, and embedded in paraffin to form solid wax blocks. The solid wax blocks were then sectioned into 5 μm-thick slices using a histological microtome (RM2016, Leica, Germany). The paraffin sections were then stained with hematoxylin and eosin (H&E) and finally sealed with neutral binder in accordance with standard histological procedures [16]. The intestinal morphology was observed using an inverted fluorescence microscope (Nikon Eclipse Ti-E, Nikon, Japan) with a 10× objective and 10× eyepiece (total magnification ×100). Sections were examined qualitatively, and no quantitative morphometric analysis of villus height or crypt depth was performed.

### 2.8. RNA Extraction and Reverse Transcription

Total RNA was extracted from intestinal samples using an RNA extraction kit (FORE GENE, Chengdu, China) following the manufacturer’s instructions. During RNA extraction, genomic DNA was removed using the DNA-cleaning column supplied with the kit prior to RNA purification. RNA concentration and purity were determined using a NanoDrop spectrophotometer (Thermo Fisher, Waltham, MA, USA). RNA integrity was assessed by agarose gel electrophoresis. Only samples with A260/A280 ratios between 1.8 and 2.0 and A260/A230 ratios > 2.0 were used for subsequent analyses. For each sample, 1 µg of total RNA was reverse-transcribed into cDNA using a ReverTra Ace qPCR RT Kit (TOYOBO, Osaka, Japan) in a total reaction volume of 20 µL, following the protocol provided by the manufacturer. The reverse-transcription reaction was performed at 37 °C for 15 min, followed by 98 °C for 5 min to inactivate the enzyme. The resulting cDNA was diluted 10-fold with nuclease-free water and stored at −20 °C until use.

### 2.9. Quantitative Real-Time PCR (qPCR)

qPCR was performed on an Applied Biosystems QuantStudio 5 system (Thermo Fisher, Waltham, MA, USA) using SYBR^®^ green real-time qPCR mix (TOYOBO). The reaction mixture (10 µL total volume) contained: 5 µL of 2× TB green premix, 0.2 µL of 10 µM forward primer, 0.2 µL of 10 µM reverse primer, 2 µL of diluted cDNA template, and 2.6 µL of RNase-free water. The cycling program was: 95 °C for 30 s (initial denaturation), followed by 40 cycles of 95 °C for 5 s and 60 °C for 30 s. A melt curve analysis was performed from 60 °C to 95 °C (increment 0.5 °C/5 s) to verify the specificity of amplification. Primer efficiencies for each gene were determined from standard curves generated by serial dilutions of pooled cDNA (five points, 10-fold dilutions). All primer pairs exhibited amplification efficiencies between 92% and 108% (R^2^ > 0.99). β-actin was used as the internal reference gene, and its expression stability across all samples was confirmed (Ct value variation < 0.5 cycles) [17]. The relative expression levels of target genes were calculated using the 2^−ΔΔCt^ method. All samples were run in triplicate. The primer sequences used are detailed in Table 2.

### 2.10. Intestinal Flora Analysis

Total microbial DNA was extracted from the pooled intestinal-content samples using a HiPure fecal DNA kit (Magen, Guangzhou, China) following the manufacturer’s instructions. The V3–V4 hypervariable region of the bacterial 16S rRNA gene was amplified by PCR using the universal primers 341F (5′-CCTACGGGNGGCWGCAG-3′) and 806R (5′-GGACTACHVGGGTWTCTAAT-3′). Each PCR reaction (50 μL) contained 5 μL of 10× KOD buffer, 5 μL of 2 mM dNTPs, 3 μL of 25 mM MgSO_4_, 1.5 μL of each primer (10 μM), 1 μL of KOD polymerase, and 100 ng of template DNA. PCR amplification was performed in triplicate for each sample. Amplicons were separated on a 2% agarose gel, purified using an AxyPrep DNA gel extraction kit (Axygen Biosciences, Union City, CA, USA), and quantified with an ABI StepOnePlus real-time PCR system (Life Technologies, Foster City, CA, USA). Purified amplicons were pooled and subjected to paired-end sequencing (2 × 250 bp) on the Illumina platform at UW Bioscience (Wuhan, China).

Bioinformatic analysis was performed following standard workflows. Raw reads were filtered using Cutadapt (v2.6) to remove primers, adapters, and low-quality sequences (sliding window of 30 bp, average quality <20, removal of reads shorter than 75% of the original length and reads containing ambiguous bases). High-quality clean reads were merged into tags using FLASH (v1.2.11) with a minimum overlap of 15 bp and a mismatch rate of 0.1. Operational taxonomic units (OTUs) were clustered at 97% sequence similarity using UPARSE in USEARCH (v11.0.667), and chimeric sequences were removed using UCHIME. Taxonomic annotation of representative OTU sequences was performed using RDP Classifier (v1.9.1) with a confidence threshold of 0.6 against the RDP 16S rRNA database (Release 19). All α-diversity, β-diversity, Venn diagram, and taxonomic composition analyses reported in this study were performed using the OTU abundance table generated by UPARSE at 97% sequence similarity.

### 2.11. Viral Challenge Test

At the end of the 8-week feeding trial, 30 unsampled fish remaining in each dietary treatment were randomly selected from the original feeding-trial cohort for the LMBV challenge. At the beginning of the 7-day challenge period, the ELE-supplemented diets were discontinued. Largemouth bass were subjected to a viral challenge test using LMBV. A preliminary test was conducted to determine the virus concentration that caused 50% mortality (LD_50_), which was found to be 2 × 10^6^ PFU/mL. Based on this, six graded concentrations of LMBV (specific concentrations determined in the pretest) were prepared. For the pretest, six fish per concentration were intraperitoneally injected with 100 µL of virus solution, and the control group received an equal volume of sterile saline. The LD_50_ was calculated using the Reed–Muench method.

For the formal challenge test, 30 fish were randomly selected from each dietary treatment group (i.e., each ELE concentration group) and evenly distributed among three replicate plastic tanks, with 10 fish per tank. Each fish was intraperitoneally injected with 100 µL of the virus solution at the predetermined LD_50_ concentration (2 × 10^6^ PFU/mL). The injected fish were then placed in plastic boxes (72 cm × 53 cm × 42 cm) with an oxygenation system. The challenge test was conducted indoors at a constant temperature of 25 °C and lasted for 7 days. Mortality was monitored every 6 h. Dead fish were promptly removed, and spleen tissues were aseptically collected for LMBV detection. LMBV infection was confirmed by detecting the LMBV major capsid protein (MCP) transcript using RT-qPCR, as described in Section 2.8 and Section 2.9. Fish showing target-specific amplification of LMBV-MCP were considered LMBV-positive. At each observation, one-third of the water was replaced with fresh, aerated water at room temperature. The cumulative mortality and survival time were recorded for each group.

At the end of the 7-day challenge period, one surviving fish was randomly selected from each replicate challenge tank, resulting in three fish per dietary treatment. The fish were anesthetized with 100 mg/L MS-222, and the spleens were aseptically excised on ice. Each spleen was processed individually without pooling, placed in a cryotube containing RNA stabilization solution, temporarily stored in liquid nitrogen, and subsequently transferred to −80 °C until RNA extraction. Total RNA extraction, genomic DNA removal, reverse transcription, and qPCR were performed as described in Section 2.8 and Section 2.9. The relative transcript levels of LMBV major capsid protein (MCP) and the inflammation-related genes IL-1β, IL-6, IL-8, and TNF-α were determined using the 2^−ΔΔCt^ method, with β-actin as the reference gene.

### 2.12. Statistical Analysis

All statistical analyses were performed using GraphPad Prism 10. Data are presented as means ± standard deviation (SD) from three independent biological replicates unless otherwise indicated. Prior to parametric analysis, data distribution was assessed using the Shapiro–Wilk test. Comparisons among multiple groups were conducted using one-way analysis of variance (ANOVA) followed by Tukey’s multiple comparison test.

## 3. Results

### 3.1. Growth Performance and Morphological Indicators

As shown in Table 3, compared with the control group (ele0), dietary supplementation with 30–120 mg/kg ELE significantly increased the specific growth rate (SGR) and supplementation with 60 and 90 mg/kg ELE significantly increased the weight gain rate (WGR) of largemouth bass (*p* < 0.05). No significant differences among the dietary treatments were observed in final average weight, survival rate, or feed conversion ratio (*p* > 0.05).

### 3.2. Hematological Parameters

**Figure 1:**
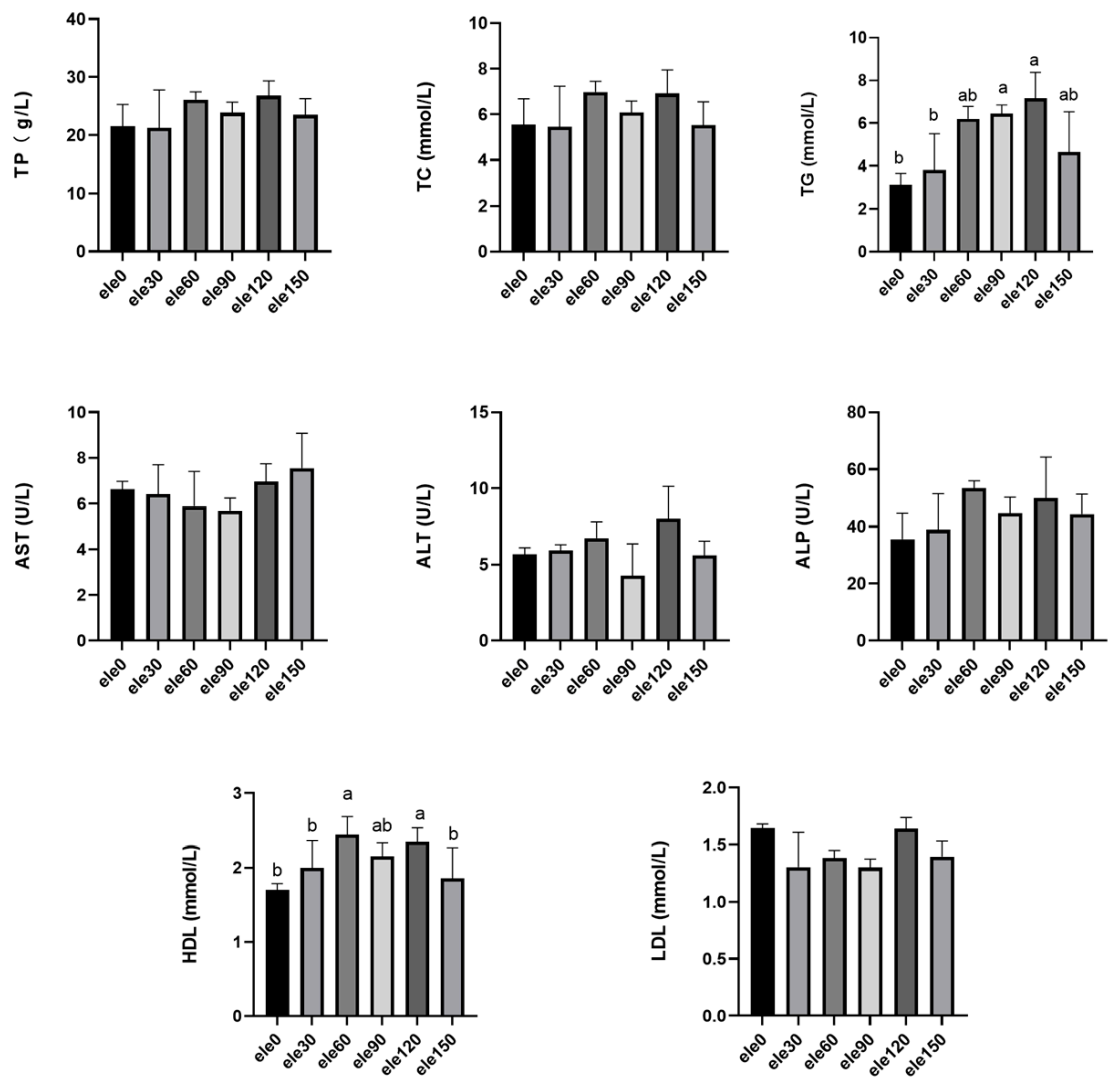
Effect of dietary supplementation of *Eucommia ulmoides* extract on hematological parameters of largemouth bass. Total protein (TP); total cholesterol (TC); triglyceride (TG); alanine aminotransferase (AST); alanine transaminase (ALT); alkaline phosphatase (ALP); high-density lipoprotein (HDL); low-density lipoprotein (LDL). Values labeled with different letters in the same figure are significantly different (*p* < 0.05).

As shown in Figure 1, compared with the control group (ele0), plasma triglyceride (TG) levels were significantly higher in the ele90 and ele120 groups, whereas plasma high-density-lipoprotein (HDL) cholesterol levels were significantly higher in the ele60 and ele120 groups (*p* < 0.05). No significant differences among the dietary treatments were observed in plasma total protein (TP), total cholesterol (TC), low-density-lipoprotein (LDL) cholesterol, aspartate aminotransferase (AST), alanine aminotransferase (ALT), or alkaline phosphatase (ALP) (*p* > 0.05).

**Figure 2:**
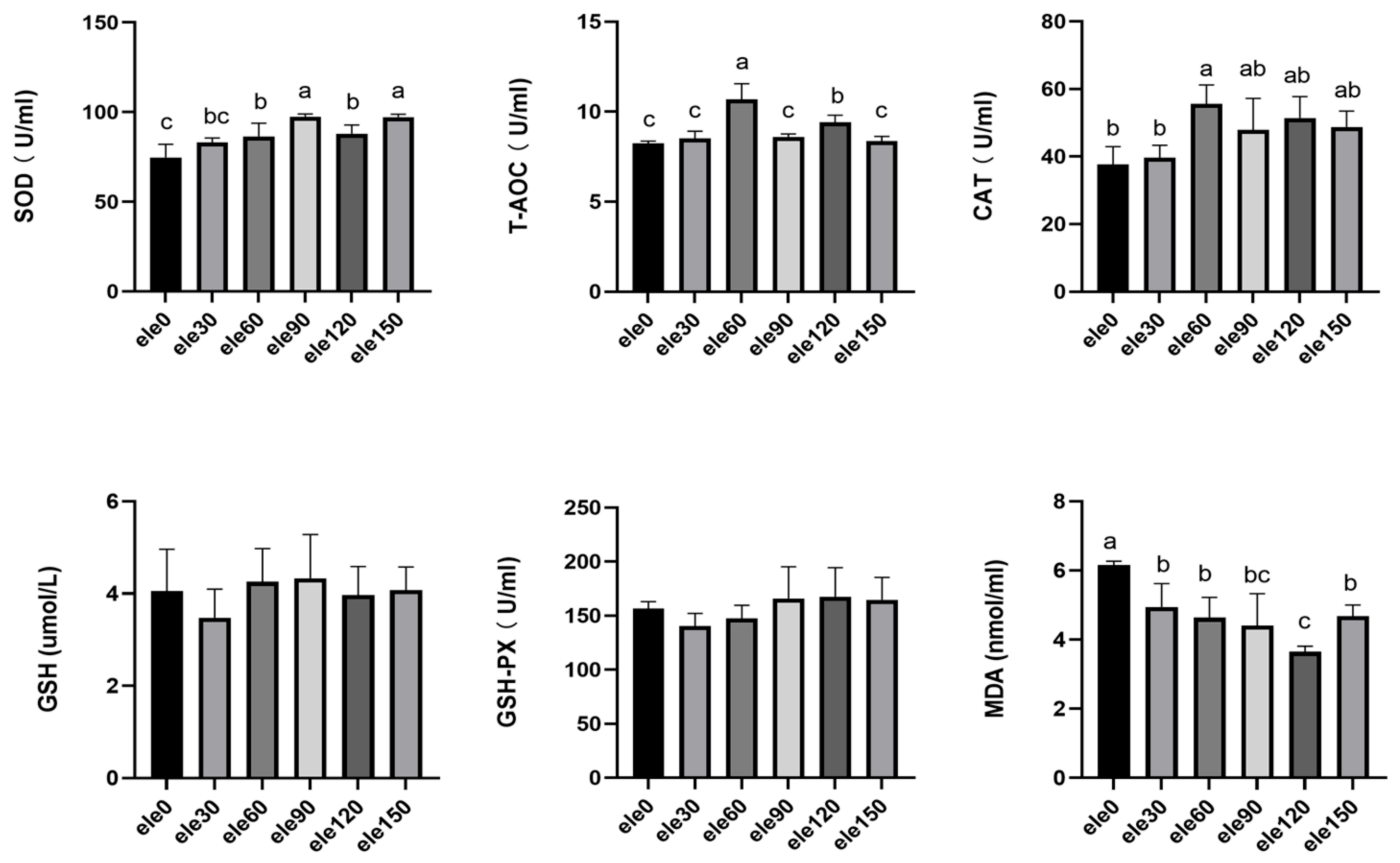
Effect of dietary supplementation of *Eucommia ulmoides* extract on plasma antioxidant enzyme activities of largemouth bass. Superoxide dismutase (SOD); total antioxidant capacity (T-AOC); catalase (CAT); glutathione (GSH); glutathione peroxidase (GSH-PX); malondialdehyde (MDA). Values labeled with different letters in the same graph are significantly different (*p* < 0.05).

As shown in Figure 2, compared with the control group (ele0), plasma SOD activity was significantly increased in the ele60, ele90, ele120, and ele150 groups, whereas no significant difference was observed between the ele30 and ele0 groups. Plasma total antioxidant capacity (T-AOC) was significantly higher in the ele60 and ele120 groups, while CAT activity was significantly higher only in the ele60 group. Plasma MDA levels were significantly lower in all ELE-supplemented groups than in the control group (*p* < 0.05). No significant differences among the dietary treatments were observed in plasma GSH levels or GSH-Px activity (*p* > 0.05).

**Figure 3:**
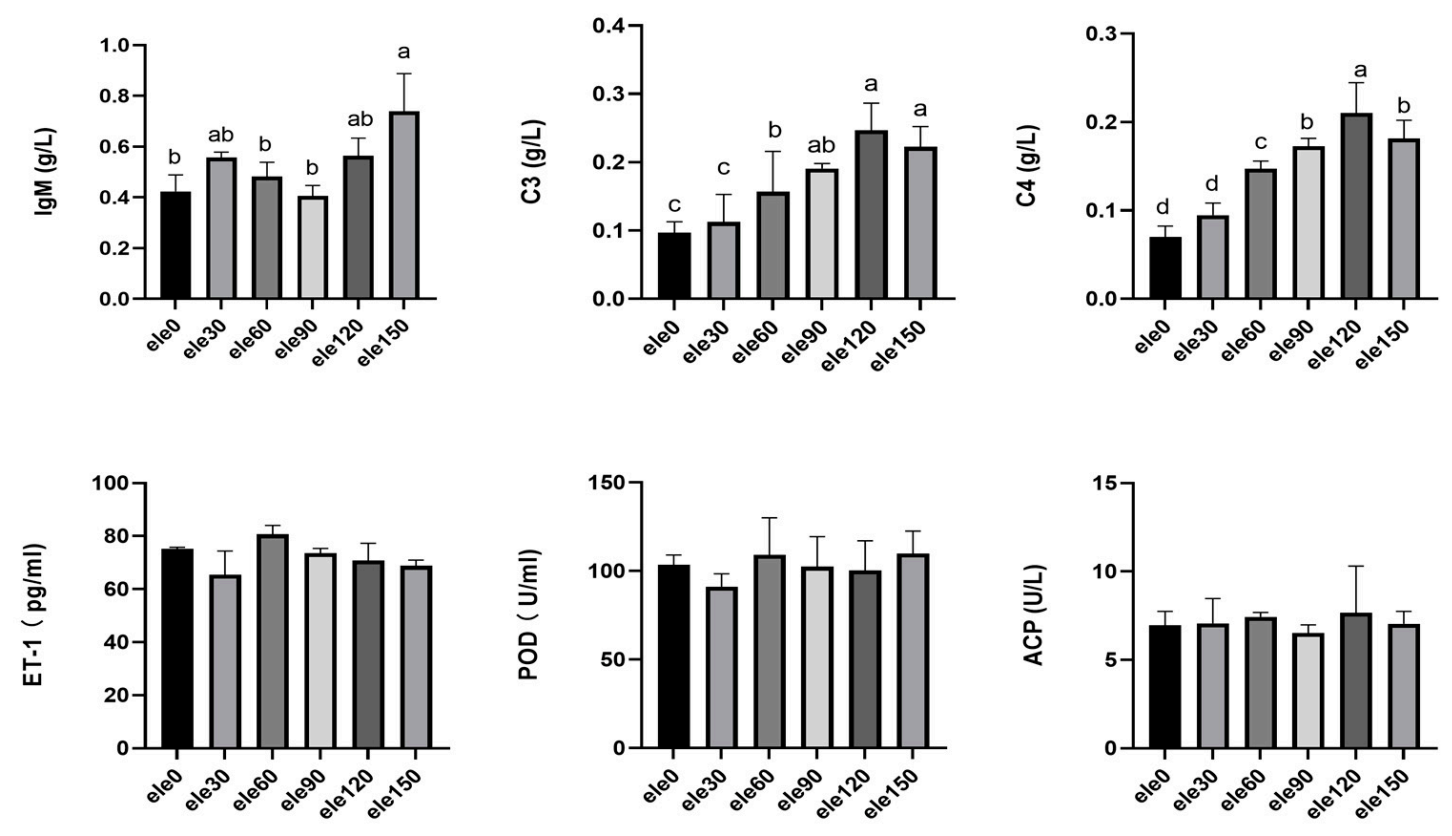
Effect of feed supplementation with *Eucommia ulmoides* extract on plasma immunoproteins activity of largemouth bass. Immunoglobulin M (IgM); complement 3 (C3); complement 4 (C4); endothelin 1 (ET-1); peroxidase (POD); acid phosphatase (ACP). Values labeled with different letters in the same graph are significantly different (*p* < 0.05).

As shown in Figure 3, ele60–ele150 significantly increased plasma C3 and C4 content (*p* < 0.05). Plasma IgM concentration was significantly higher in the ele150 group than in the ele0, ele60, and ele90 groups (*p* < 0.05). The ele30 and ele120 groups showed intermediate values and did not differ significantly from either the ele0 or ele150 group.

### 3.3. Intestinal Antioxidant Capacity

**Figure 4:**
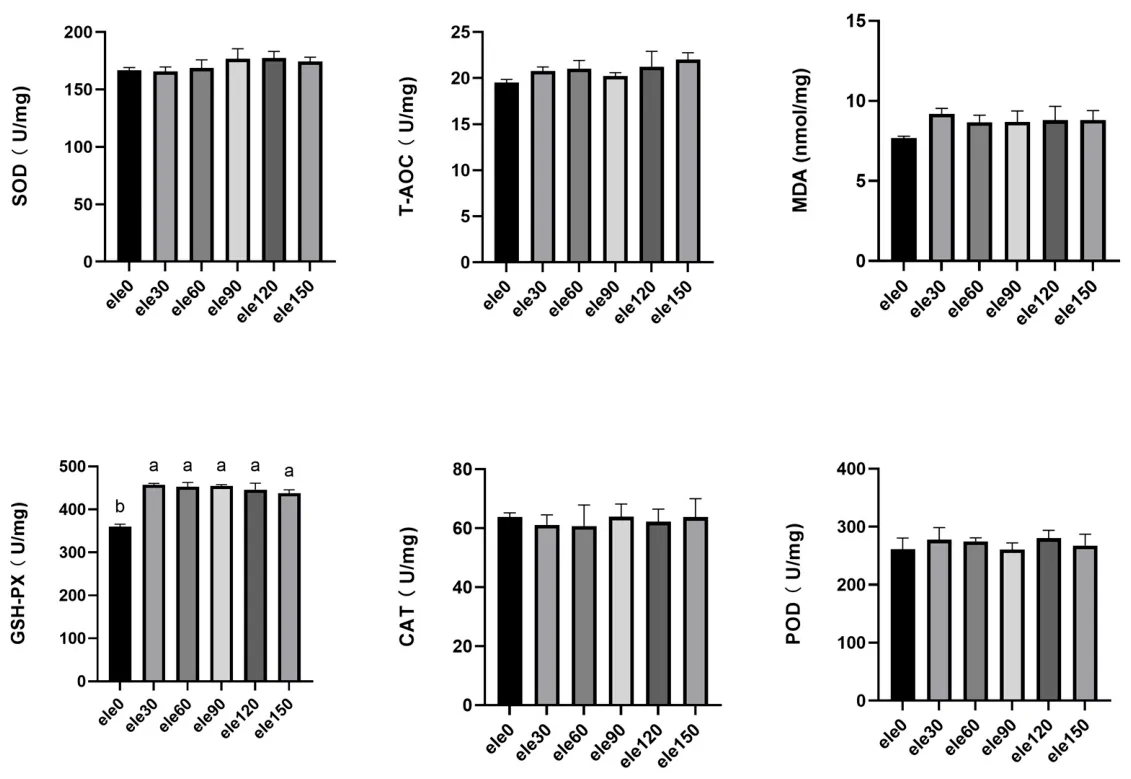
Effect of feed supplementation with *Eucommia ulmoides* extract on the activity of intestinal antioxidant enzymes in largemouth bass. Superoxide dismutase (SOD); total antioxidant capacity (T-AOC); catalase (CAT); peroxidase (POD); glutathione peroxidase (GSH-PX); malondialdehyde (MDA). Values labeled with different letters in the same graph are significantly different (*p* < 0.05).

As shown in Figure 4, ele30–ele150 significantly increased intestinal GSH-PX activity (*p* < 0.05). In addition, the addition of *Eucommia ulmoides* extract to the feed had no significant effect on plasma T-AOC, MDA content, or SOD, CAT, and POD activities (*p* > 0.05).

**Figure 5:**
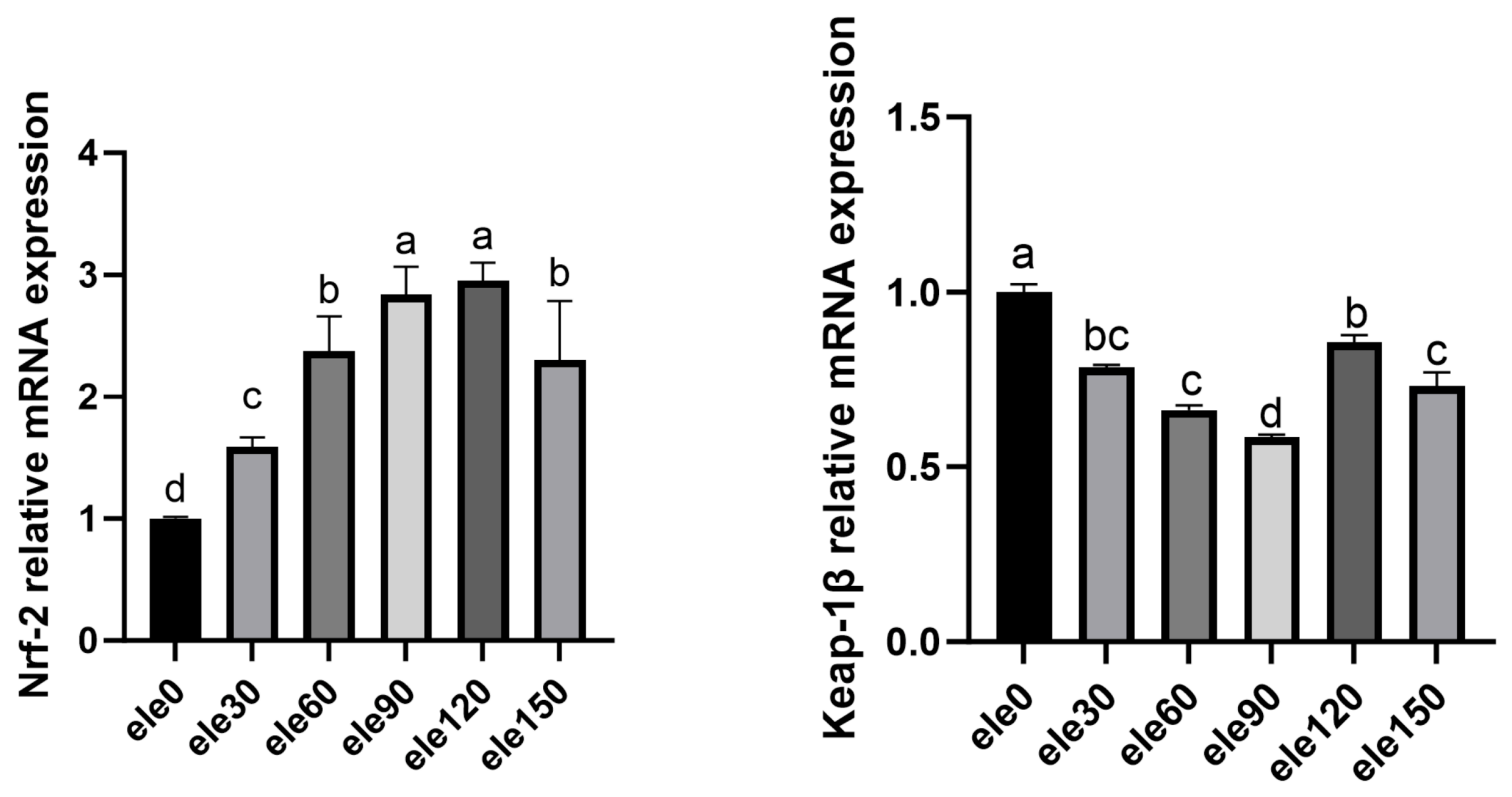
Effect of feed supplementation with *Eucommia ulmoides* extract on the expression of intestinal antioxidant genes in largemouth bass. Nuclear factor E2-associated factor 2 (Nrf2); Kelch-like ECH-associated protein 1 (Keap1). Values labeled with different letters in the same figure are significantly different (*p* < 0.05).

As shown in Figure 5, compared with the control group (ele0), intestinal Nrf2 mRNA expression was significantly higher and Keap1β mRNA expression significantly lower in all ELE-supplemented groups (*p* < 0.05). The magnitude of these expression changes differed among the ELE inclusion levels.

### 3.4. Intestinal Inflammatory Response

**Figure 6:**
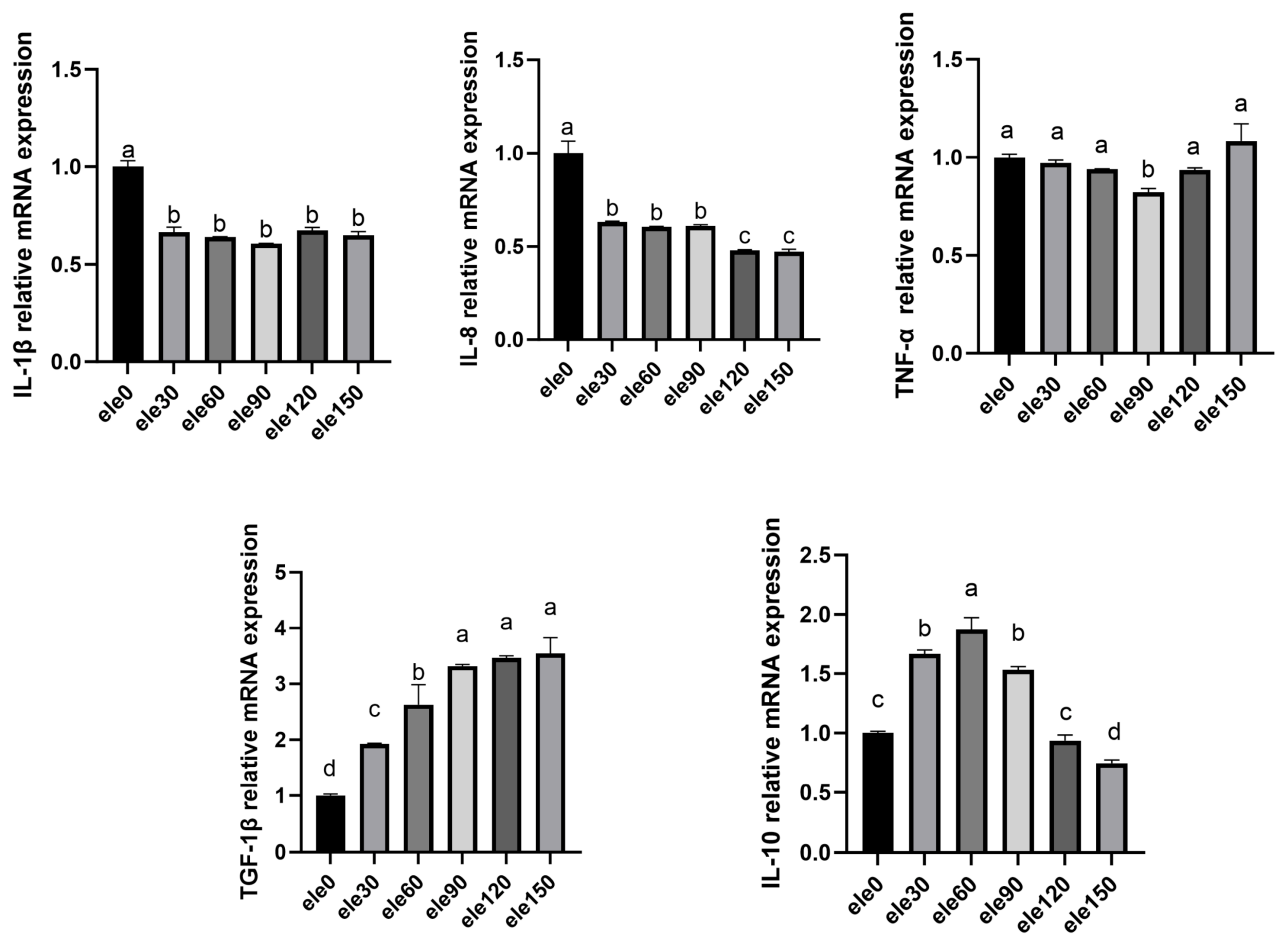
Effect of feed supplementation with *Eucommia ulmoides* extract on the expression of intestinal inflammatory factor genes in largemouth bass. Tumor necrosis factor α (TNF-α); interleukin 8 (IL-8); interleukin 1β (IL-1β); transforming growth factor β (TGF-β); interleukin 10 (IL-10). Values marked by different letters in the same figure are significantly different (*p* < 0.05).

As shown in Figure 6, ele30–ele150 significantly inhibited (*p* < 0.05) the expression of the intestinal proinflammatory factors IL-8 and IL-1β. The addition of 90 mg/kg of ELE significantly inhibited the expression of intestinal proinflammatory factor TNF-α (*p* < 0.05). In addition, ele30–ele150 significantly promoted the expression of the intestinal anti-inflammatory cytokine factor TGF-β, and ele30–ele90 significantly promoted the expression of the intestinal anti-inflammatory factor IL-10 (*p* < 0.05).

### 3.5. Intestinal Cell Apoptosis

**Figure 7:**
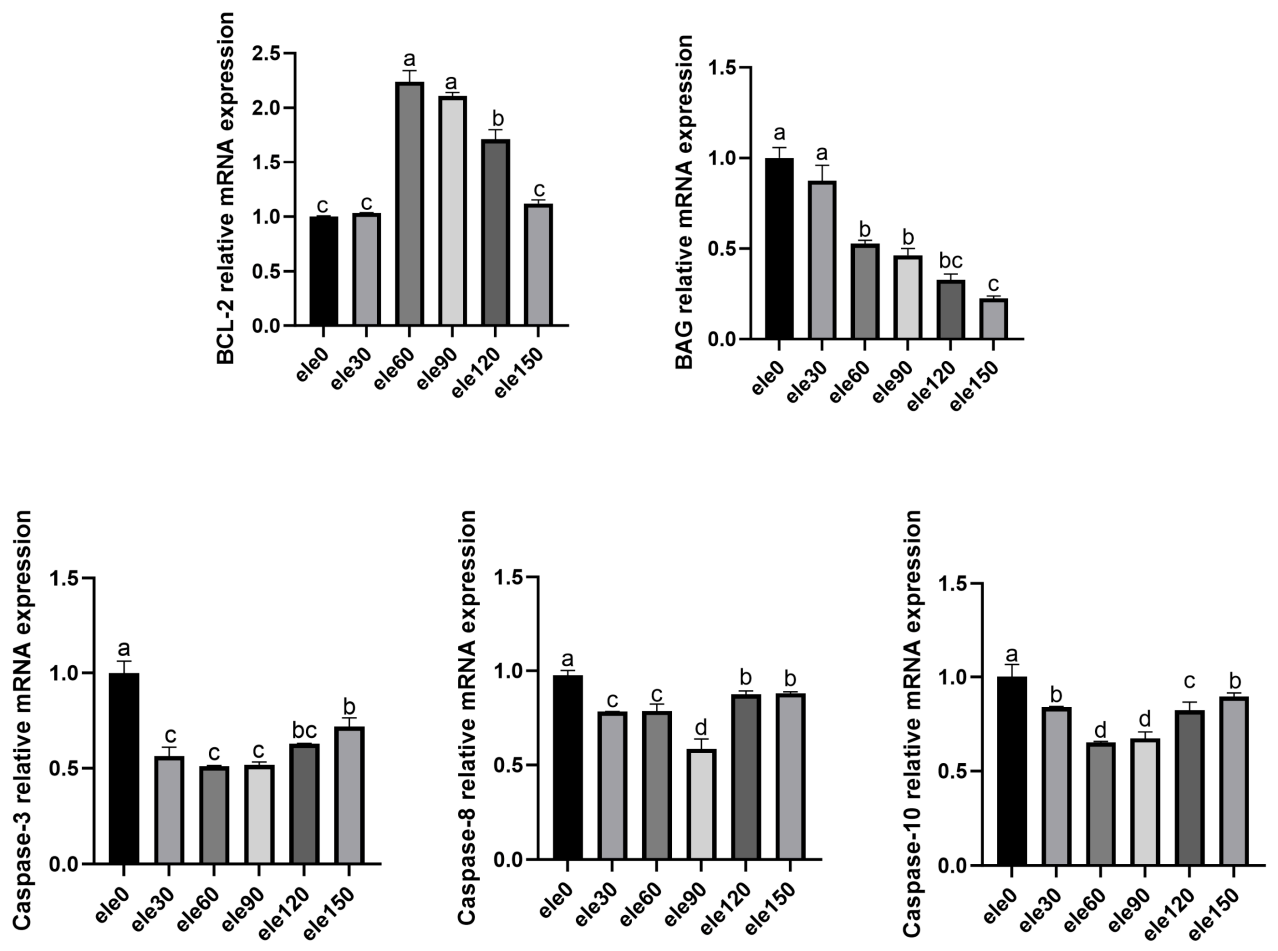
Effect of feed supplementation with *Eucommia ulmoides* extract on the expression of intestinal apoptotic factor genes in largemouth bass. B-cell lymphoma 2 (BCL-2); Bcl-2-associated anti-apoptotic gene protein family 8 (BAG); cysteinyl asparagine 3 (caspase 3); cysteinyl asparagine 8 (caspase 8); cysteinyl asparagine 10 (caspase 10); Values labeled with different letters in the same figure are significantly different (*p* < 0.05).

As shown in Figure 7, compared with the control group (ele0), intestinal BCL-2 mRNA expression was significantly higher in the ele60, ele90, and ele120 groups, whereas BAG mRNA expression was significantly lower in the ele60, ele90, ele120, and ele150 groups (*p* < 0.05). The mRNA expression levels of the apoptosis-related genes caspase 3, caspase 8, and caspase 10 were significantly lower in all ELE-supplemented groups than in the control group (*p* < 0.05). Among the ELE treatments, the lowest caspase 10 expression levels were observed in the ele60 and ele90 groups.

### 3.6. Intestinal Barrier Proteins

**Figure 8:**
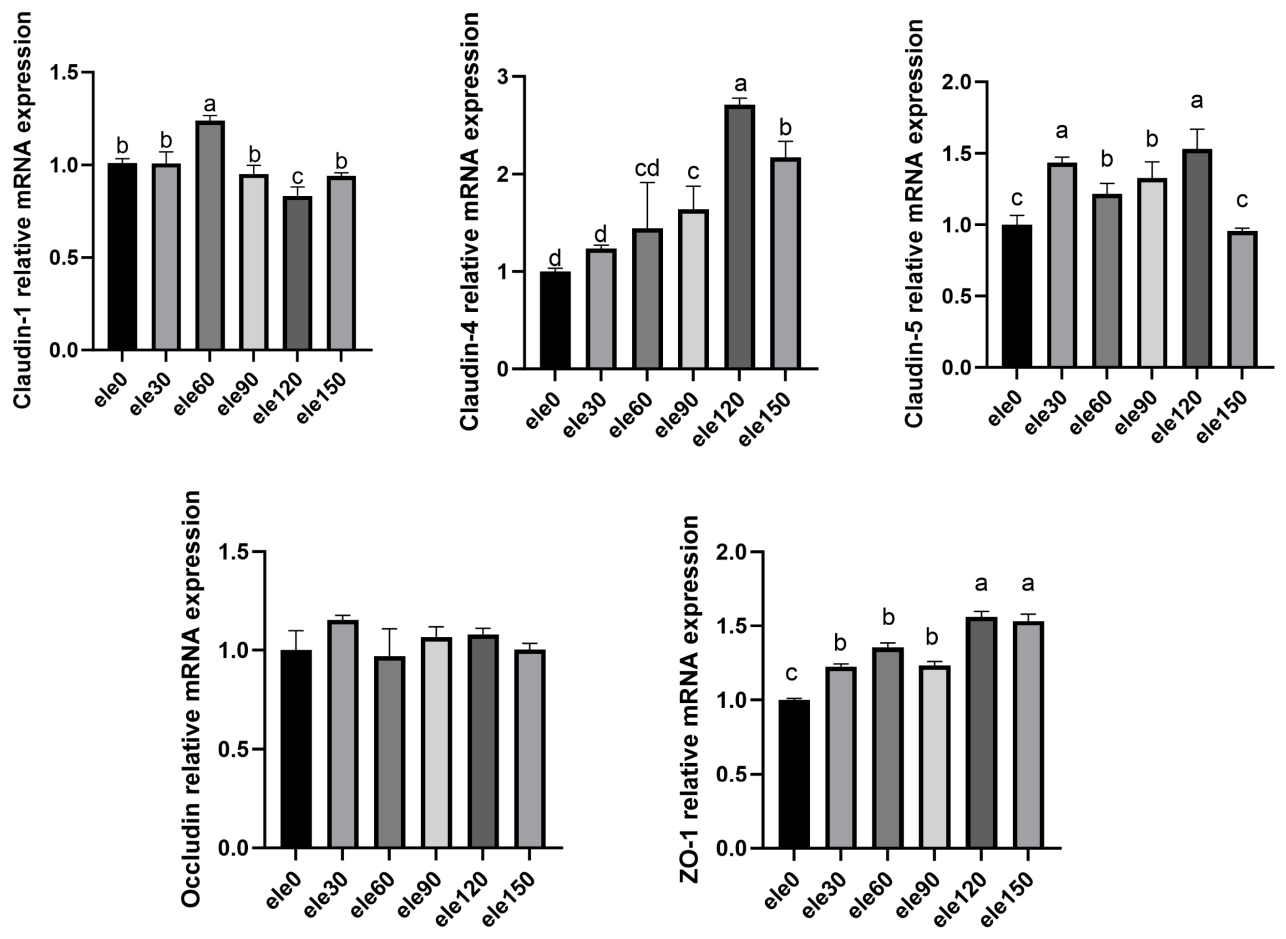
Effect of dietary supplementation with *Eucommia ulmoides* extract on intestinal tight-junction protein gene expression in largemouth bass. Zonula occludin 1 (ZO-1). Values labeled with different letters in the same figure are significantly different (*p* < 0.05).

As shown in Figure 8, the relative expression level of intestinal claudin 1 was significantly increased by the ele60, while the relative expression level of intestinal ZO-1 and claudin 4 was significantly increased by the ele120–ele150 (*p* < 0.05). The relative expression levels of intestinal claudin 5 were significantly (*p* < 0.05) increased by ele30–ele120. In addition, the addition of ELE to the feed had no significant effect on the relative expression level of intestinal occludin (*p* > 0.05).

### 3.7. Intestinal Histomorphology

**Figure 9:**
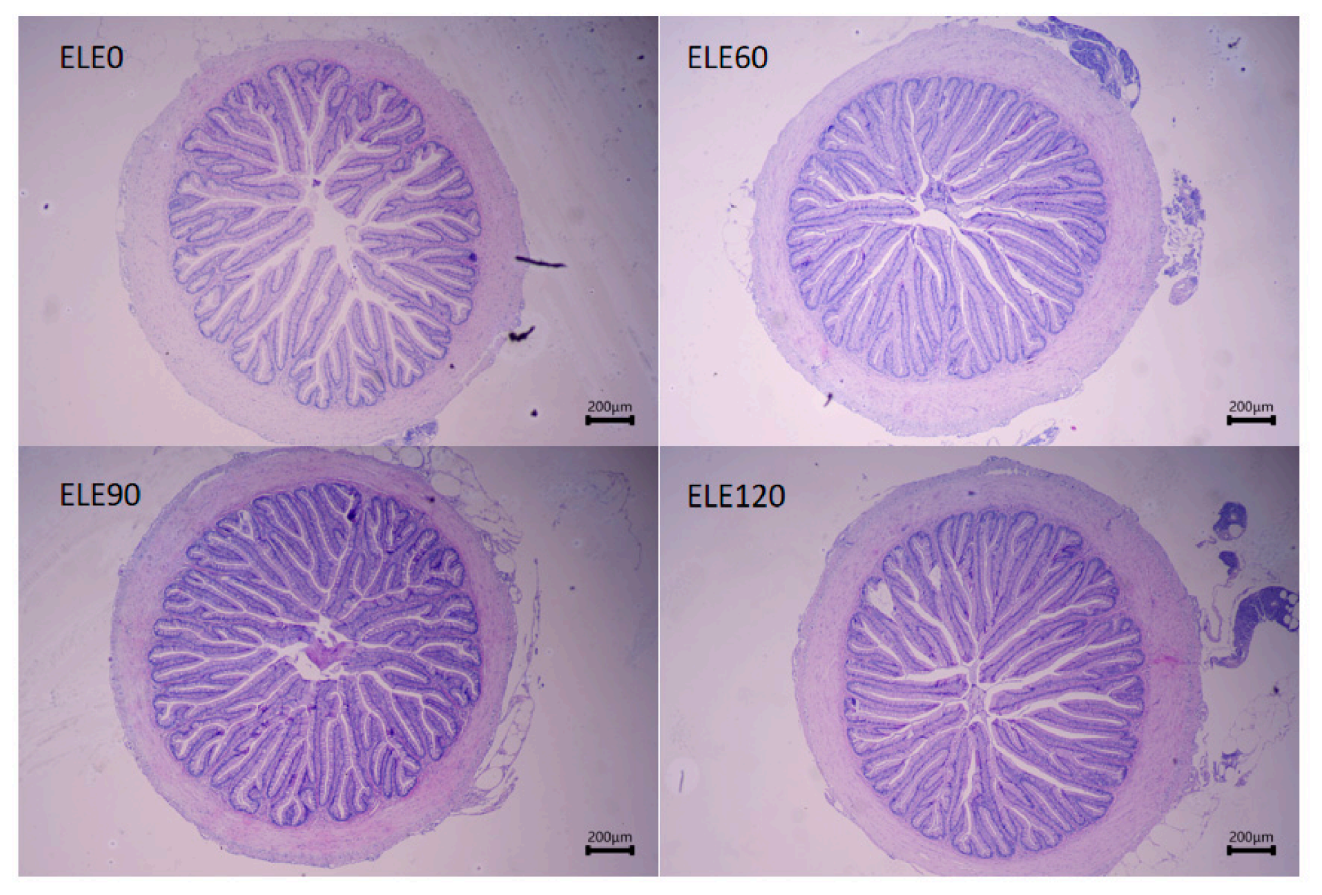
Effect of dietary supplementation with *Eucommia ulmoides* extract of largemouth bass on low-magnification H&E staining images.

Representative H&E-stained intestinal sections are shown in Figure 9. At low magnification, all examined groups exhibited a generally preserved intestinal wall and clearly recognizable mucosal folds, without obvious large-scale disruption of the tissue architecture. Descriptively, the mucosal folds in the ele60 and ele90 groups appeared more elongated, densely distributed, and regularly arranged than those in the ele0 group, whereas the ele120 group retained an overall mucosal architecture comparable to that observed in the other ELE-supplemented groups.

### 3.8. Intestinal Bacteria Analysis

**Figure 10:**
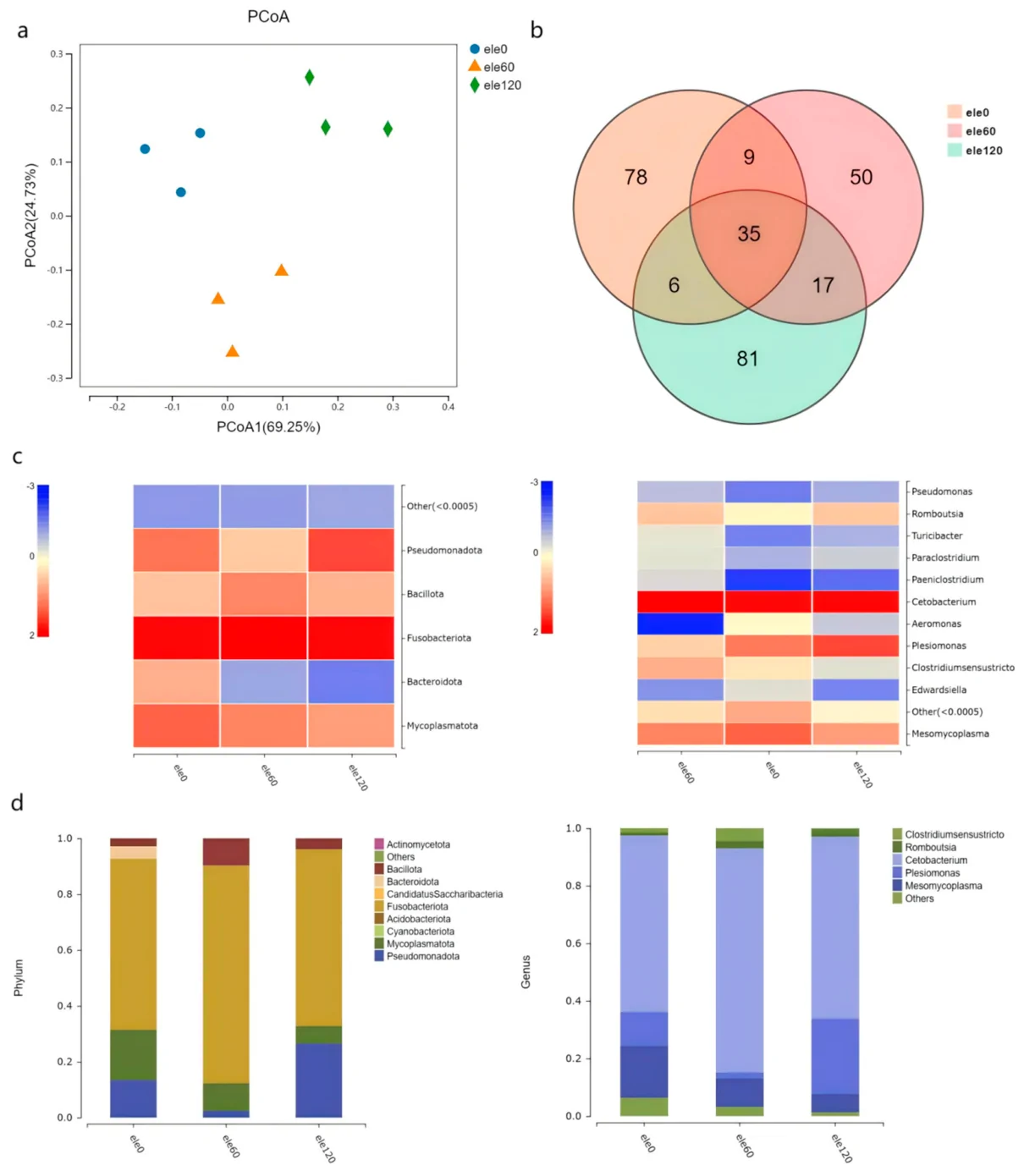
Descriptive analysis of the gut microbiota of largemouth bass fed diets containing different levels of ELE. (**a**) Principal component analysis; (**b**) Venn diagram showing shared and unique OTUs; (**c**) heatmap of microbial composition; (**d**) stacked bar plots showing the relative abundance of the predominant taxa. PCA separation and differences in taxonomic relative abundance are presented descriptively because no PERMANOVA or differential abundance analysis was performed.

Gut microbiota is an important indicator of gut health. Gut microbial communities were evaluated using 16S rRNA gene sequencing, including analyses of α-diversity, β-diversity, and taxonomic composition. As shown in Table 4, the Shannon index was significantly lower in the ele120 group than in the ele0 group (*p* < 0.05), whereas the ele60 group did not differ significantly from the control. No significant differences among the three groups were observed in the Simpson index or the number of OTUs. The PCA plot showed that samples from the ele0, ele60, and ele120 groups occupied partially separated positions in the ordination space (Figure 10a). However, because no PERMANOVA or other formal statistical test of β-diversity was performed, this pattern was interpreted descriptively. The Venn diagram in Figure 10b illustrates the shared and unique OTUs among the three groups, while the heatmap and stacked bar plots in Figure 10c,d show descriptive variation in microbial composition and relative abundance. The predominant phyla were Fusobacteriota, Mycoplasmatota, Pseudomonadota, and Bacillota, and the predominant genera included Cetobacterium, Mesomycoplasma, Plesiomonas, and Romboutsia. The relative abundance profiles of Mesomycoplasma and Plesiomonas, as well as those of Acidobacteriota and Mycoplasmatota, differed descriptively among the dietary treatments. Because differential abundance analysis was not performed, these apparent differences were not interpreted as statistically significant.

### 3.9. LMBV Infection

**Figure 11:**
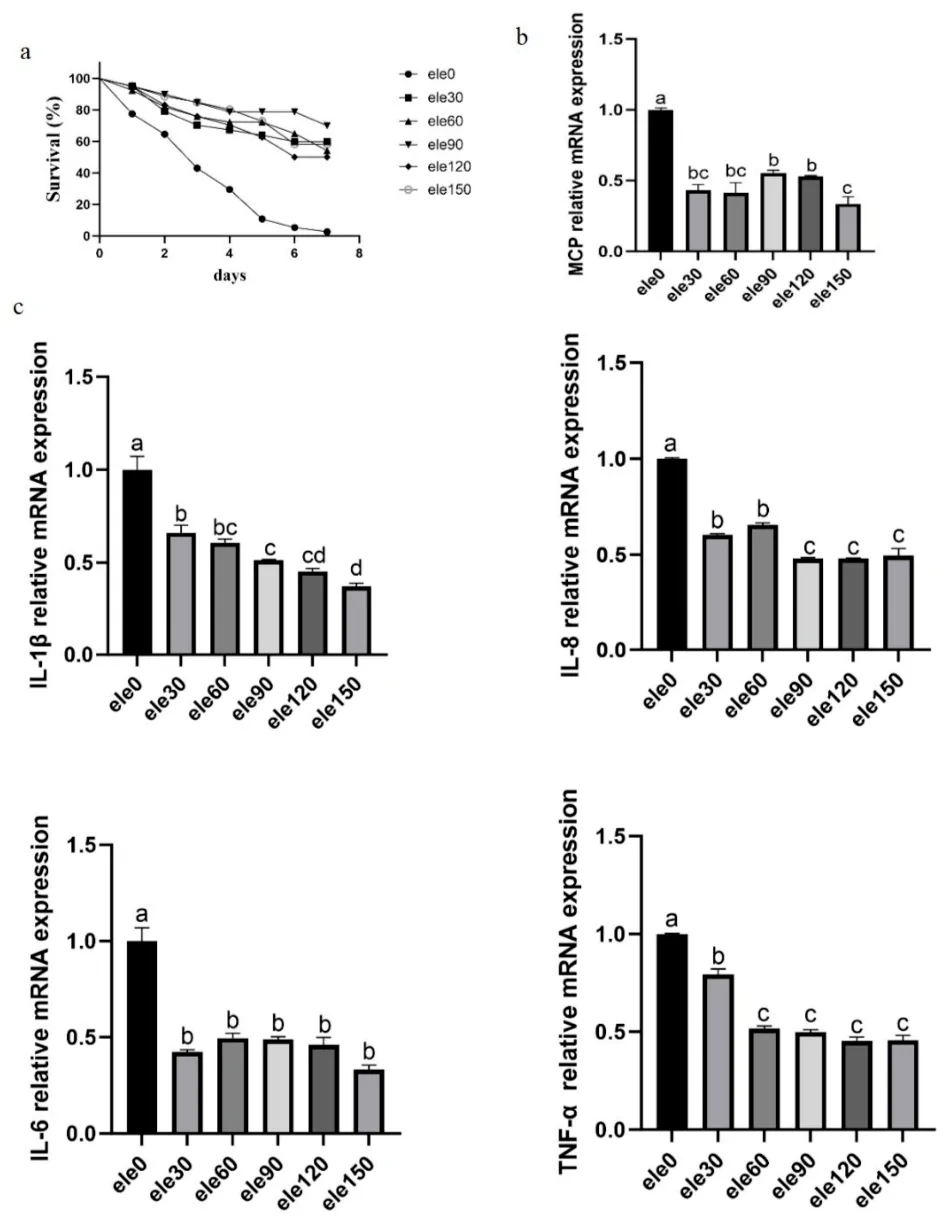
Protective effect of dietary supplementation of *Eucommia ulmoides* extract on largemouth bass following LMBV infection. (**a**) Survival rate 7 d after LMBV infection; (**b**) viral MCP expression; (**c**) proinflammatory factor gene expression. Values labeled with different letters in the same figure are significantly different (*p* < 0.05).

In the antiviral experiments, the control group showed rapid death from the first day, while the ELE group showed some protection, with a gradual decrease in death from the fourth day of infection until the seventh day (Figure 11a). Among the ELE-supplemented treatments, the ele90 group, corresponding to 90 mg/kg ELE, showed the highest day-7 survival rate of 70.0%, compared with 3.3% in the ele0 group. By detecting LMBV-MCP gene expression in the spleen tissue of largemouth bass after infection, it was found that the ELE group significantly reduced the expression level of MCP (Figure 11b), suggesting that it might have inhibited the replication function of the virus. In addition, the results of the spleen inflammatory factor-related genes showed that the ELE significantly reduced the expression of proinflammatory factors, thus alleviating the inflammatory response of largemouth bass after infection (Figure 11c). These results suggest that ELE may protect largemouth bass from LMBV infection by inhibiting viral replication.

## 4. Discussion

Growth performance, encompassing metrics such as feed conversion ratio, weight gain rate, and specific growth rate, is a key indicator for evaluating feed efficacy in fish farming [18]. Studies show that dietary supplementation with Eucommia extract enhances the growth performance of grass carp [19], and large yellow croaker [8] by significantly increasing intestinal digestive enzyme activity and protein digestibility. In the present study, however, the response was selective rather than uniform: SGR was higher in the ele30–ele120 groups and WGR was higher in the ele60 and ele90 groups, whereas FAW, FCR, and SR did not differ significantly among treatments. Accordingly, the results support a modest improvement in selected growth indices at intermediate ELE inclusion levels, but do not demonstrate a generalized enhancement of growth or feed utilization. Because nutrient digestibility and digestive enzyme activity were not measured, the physiological basis of the SGR and WGR responses cannot be determined from the present data.

Plasma biochemical indicators are vital in fish research, serving as key reflections of physiological status, health, and environmental responses [20]. Analysis of plasma enzyme activity, metabolic products, and ion concentrations can reveal disease, malnutrition, or other health issues in fish [21]. For instance, elevated ALT and AST levels suggest liver damage, whereas abnormal urea and creatinine levels indicate kidney dysfunction [22]. Plasma immunoglobulins (e.g., IgM and IgT) specifically recognize and bind pathogens. The resulting antigen–antibody complexes mediate enhanced phagocytosis and clearance by phagocytes [23]. Furthermore, activation of the complement system (e.g., C3, C4) enhances antibacterial, antiviral, and lytic activity, strengthening pathogen resistance in fish [24]. In the present study, dietary ELE supplementation was associated with changes in several plasma biochemical, antioxidant, and immune-related indices. Plasma TG and HDL levels were higher at selected inclusion levels, whereas ALT and AST activities were not significantly affected. Plasma SOD activity was higher in the 60–150 mg/kg groups, while significant increases in T-AOC and CAT activity were observed only at selected intermediate inclusion levels. In addition, plasma MDA concentrations were lower in all ELE-supplemented groups than in the control group. Although MDA concentrations tended to increase again at the higher inclusion levels after reaching the lowest value at an intermediate level, they remained below the control value. Therefore, this pattern may indicate a non-linear antioxidant response or a reduced marginal benefit at higher inclusion levels, but it does not provide sufficient evidence that high ELE doses induced oxidative stress. Analogous research demonstrated that dietary supplementation of 10 g/kg Eucommia leaf extract significantly elevated T-AOC and CAT activity in yellow croaker [8]. In another study, supplementation with 4 g/kg Eucommia bark and leaf extract significantly increased SOD and GSH-Px activity while reducing MDA levels in grass carp muscle [19]. In this study, plasma C3 and C4 concentrations were also higher in several ELE-supplemented groups, and IgM was highest in the 150 mg/kg group. However, these responses did not follow a consistent monotonic dose–response pattern. Collectively, the observed changes suggest that ELE may influence selected antioxidant and immune-related indices in largemouth bass, particularly at intermediate inclusion levels, but these indices alone are insufficient to demonstrate a generalized enhancement of immune function or disease resistance.

Intestinal immune function plays a vital role in maintaining gut health in largemouth bass [25]. Functioning as a vital immune organ, the gut therefore plays an indisputable role in determining overall health status [26]. This study found that expression analysis of intestinal immune factors revealed a significant suppression of the proinflammatory factors IL-8 and IL-1β in all groups supplemented with Eucommia extract. Notably, expression of the proinflammatory factor TNF-α was significantly downregulated in the 90 mg/kg ELE group relative to the control. Previous research indicates that chlorogenic acid from *Eucommia ulmoides* functions as an anti-inflammatory agent by downregulating CD14, thereby blocking the NF-κB pathway and inhibiting P65 phosphorylation, which ultimately reduces the expression of TNF-α, IL-1β, and IL-6 in LPS-stimulated intestinal epithelial cells [27]. Consistent with these findings, the changes in inflammation-related gene expression observed in the present study suggest that dietary ELE may contribute to the modulation of intestinal inflammatory responses in largemouth bass. However, because only selected cytokine transcripts were measured, these results do not demonstrate a generalized enhancement of intestinal immune function.

Oxidative stress induces lipid peroxidation, protein oxidation, and DNA damage in intestinal cells, which in turn compromises intestinal barrier integrity and function [28,29]. Antioxidant enzymes like SOD and CAT protect intestinal cells from oxidative damage by neutralizing free radicals, thereby preserving normal intestinal function [30]. The Nrf2/Keap1 pathway modulates downstream targets to induce the expression of antioxidant genes and enhance enzymatic activity, ultimately preventing cellular oxidative stress [31]. As a highly conserved transcription factor, Nrf2 plays a central role in antioxidant defense by binding to and activating the antioxidant response element [32]. Keap1 is a key negative regulator of Nrf2 [33]. The Keap1/Nrf2 pathway maintains redox homeostasis through two-step regulation. Normally, Keap1 mediates the constitutive ubiquitination and proteasomal degradation of Nrf2. Under oxidative insult, this degradation is halted, allowing Nrf2 to accumulate and translocate into the nucleus. Consequently, Nrf2 activates the ARE, promoting the transcription of a battery of cytoprotective and antioxidant enzymes [34]. Additionally, studies show that Eucommia extract improves intestinal structure in diabetic osteoporosis (DOP) model mice via activation of the Nrf2/HO-1 pathway [35]. Studies indicate that by supplementing the diet with Eucommia extract, the mRNA levels of Nrf2 and its downstream antioxidant genes (e.g., CAT and SOD) are thereby upregulated in tilapia intestines [36]. In the present study, dietary ELE supplementation significantly increased intestinal GSH-Px activity, whereas SOD, CAT, T-AOC, and MDA were not significantly affected. ELE supplementation was also associated with higher intestinal Nrf2 mRNA expression and lower Keap1β mRNA expression than the control group, although the magnitude of these transcriptional changes varied among inclusion levels. These expression profiles are consistent with the possible involvement of Nrf2/Keap1β-related redox regulation but do not directly demonstrate activation of this signaling pathway, because Nrf2 protein abundance, nuclear translocation, and downstream protein expression or activity were not examined. Although some responses were less pronounced at the higher ELE inclusion levels, no dose–response or breakpoint analysis was performed; therefore, the present data do not identify a threshold dose or inflection point. A biphasic response could theoretically be associated with hormetic or pro-oxidant effects of polyphenolic compounds at high concentrations. However, the unchanged intestinal MDA concentration and the consistently higher GSH-Px activity in the ELE-supplemented groups do not provide direct evidence of high-dose-induced oxidative stress or impaired intestinal antioxidant capacity. Thus, the less pronounced responses at higher inclusion levels should be interpreted cautiously as a possible plateau or non-linear response. Further studies incorporating additional dose levels, direct oxidative-damage measurements, Nrf2 protein abundance and nuclear translocation, and downstream target activity are required to determine whether a true biphasic response exists and to clarify the involvement of Nrf2/Keap1β signaling.

Apoptosis serves as an essential regulatory mechanism for intestinal cellular homeostasis and epithelial turnover [37]. Apoptosis ensures intestinal tissue health and stability by eliminating senescent or damaged cells [38,39]. Previous studies have shown that *Eucommia* extract suppresses TLR2-mediated apoptosis in tilapia through the downregulation of FADD mRNA expression [36]. In the present study, intestinal BCL-2 mRNA expression was higher in the 60–120 mg/kg groups, whereas BAG mRNA expression was lower in the 60–150 mg/kg groups. The mRNA expression levels of caspase 3, caspase 8, and caspase 10 were also lower in the ELE-supplemented groups than in the control group. These transcriptional changes suggest that dietary ELE may modulate apoptosis-related processes in the intestine. However, because apoptotic cell abundance, caspase activity, and apoptosis-related protein expression were not measured, the present results do not directly demonstrate inhibition of intestinal cell apoptosis. Tight-junction proteins, including occludin, claudins, and ZO-1, contribute to the regulation of intestinal epithelial permeability and barrier integrity [40]. In this study, the expression responses of tight-junction-related genes varied according to the gene and ELE inclusion level: claudin 1 mRNA expression was higher in the 60 mg/kg group, ZO-1 and claudin 4 expression was higher in the 120–150 mg/kg groups, and claudin 5 expression was higher in the 30–120 mg/kg groups, whereas occludin expression was unaffected. Chlorogenic acid has similarly been reported to alter the expression of claudin 1, occludin, and ZO-1 in LPS-challenged Caco-2 cells [41]. Collectively, the observed transcriptional profiles suggest that ELE may influence apoptosis- and barrier-related regulation in the intestine. Nevertheless, these gene-expression changes alone do not establish inhibition of apoptosis or improvement of intestinal barrier function, which would require confirmation through protein-level, histological, apoptosis, and intestinal permeability measurements.

The gut microbiota of fish encompasses a diverse array of microorganisms, including bacteria, archaea, fungi, yeasts, viruses, and protozoa. Its formation is shaped by multiple factors ranging from host genetics and dietary composition to environmental conditions and seasonal variations [42]. The gut microbiota is integral to fish nutrition, facilitating the digestion and absorption of dietary components [43]. In the present study, descriptive analysis indicated differences in bacterial diversity and community composition among the ele0, ele60, and ele120 groups. At the phylum level, Fusobacteriota remained dominant across the three groups, whereas the relative abundance of Acidobacteriota and several other low-abundance phyla varied among treatments. At the genus level, Cetobacterium was the predominant taxon in all three groups and showed numerically higher relative abundance in the ele60 group, while Romboutsia also appeared relatively more abundant at this inclusion level. In contrast, the relative abundance of Mesomycoplasma was numerically lower with increasing ELE inclusion, whereas Plesiomonas showed different patterns at 60 and 120 mg/kg. Beyond their taxonomic abundance, Cetobacterium and Romboutsia may have potential metabolic relevance in fish. Certain Cetobacterium, particularly C. somerae, can produce acetate and vitamin B12 and have been associated with carbohydrate utilization and metabolic regulation in fish [44]. Similarly, some Romboutsia strains possess carbohydrate-fermenting capacity and can produce short-chain fatty acids, suggesting a potential contribution to nutrient metabolism [45]. Thus, the numerical enrichment of Cetobacterium and Romboutsia in the ele60 group could be consistent with a possible shift in microbial carbohydrate fermentation or metabolite-producing potential. However, the present study did not measure short-chain fatty acids, vitamin B12, microbial genes, or metabolic pathways, and genus-level relative abundance alone cannot establish the metabolic activity or health effects of these taxa. Likewise, changes in the relative abundance of Mesomycoplasma, Plesiomonas, and Acidobacteriota do not establish whether these taxa were beneficial, harmful, or directly involved in host health. Bacterial pathogenicity and intestinal invasion were not assessed. Therefore, the observed microbial differences should be interpreted only as compositional changes rather than evidence that ELE suppressed harmful bacteria, prevented pathogen invasion, or improved intestinal function through microbial metabolites. Because only three dietary groups were analyzed and no formal statistical testing of individual taxonomic abundance or β-diversity was performed, these findings remain descriptive and require further validation through metagenomic, metabolomic, and short-chain fatty-acid analyses.

Viruses are prolific in aquatic environments, particularly in high-density aquaculture settings [46]. LMBV is a highly pathogenic agent that afflicts largemouth bass, often leading to high mortality and substantial economic damage in aquaculture [47]. Furthermore, studies have shown that LMBV infection upregulates the expression of proinflammatory cytokines in largemouth bass [48]. Furthermore, it has been reported that in response to infection by the H1N1 influenza virus, ELE can markedly suppress the upregulation of proinflammatory mediators [49]. In the present challenge experiment, fish previously fed the 90 mg/kg ELE diet showed the highest observed day-7 survival rate of 70.0%, compared with 3.3% in the control group. At 7 dpi, ELE-supplemented fish also showed lower splenic LMBV-MCP mRNA abundance and lower expression of selected proinflammatory genes. Reduced MCP transcript abundance may be consistent with lower viral transcriptional activity or viral burden; however, it does not directly demonstrate inhibition of viral replication or infectivity. Similarly, the inflammatory gene-expression profiles obtained at a single endpoint reflect the host response at 7 dpi but cannot establish the temporal sequence or mechanism underlying the survival differences. Because spleen samples were collected at only one post-challenge time point and gene expression during viral infection is highly dynamic, the present findings should be interpreted as an association between prior ELE supplementation and the observed post-challenge responses rather than evidence of a defined antiviral mechanism. Serial sampling combined with measurements of viral genome copy number, infectious viral titers, and protein-level immune responses would be required to determine whether ELE directly affects LMBV replication or primarily modulates the host response.

## 5. Conclusions

Dietary ELE supplementation produced inclusion-level-specific responses in juvenile largemouth bass, with 60–90 mg/kg showing the most consistent favorable effects on selected growth, antioxidant, immune-related, and intestinal indices. ELE also altered the mRNA expression of genes associated with redox regulation, inflammation, apoptosis, and tight junctions; however, these transcriptional changes do not directly establish pathway activation or corresponding functional improvements. The microbiota results indicated descriptive changes in bacterial community composition. Following LMBV challenge, the 90 mg/kg group showed the highest observed day-7 survival rate, accompanied by lower splenic LMBV-MCP and proinflammatory gene expression. These findings suggest a potential protective association, but do not demonstrate direct inhibition of viral replication. Overall, 60–90 mg/kg ELE appears to be a promising inclusion range, although further functional and mechanistic validation is required.

## Figures and Tables

**Table 1:** Ingredients and nutritional composition of the basal diet (%).

Ingredients	Percentage
White fish meal	58
Soybean meal ^c^	9
Wheat flour	10
Corn gluten meal	10
Soybean oil	3.25
Fish oil	3.25
Ca(H_2_PO_4_)_2_	1.5
Choline chloride	0.5
Compound premix ^a^	3
Carboxymethyl cellulose	0.5
Lecithin	1
Total	100.0
Proximate composition	
Dry material (%)	89.3
Crude protein (%, DM)	47.5
Crude lipid (%, DM)	12.3
Ash (%, DM)	10.4
Gross energy ^b^ (MJ/kg)	17.9

^a^ The vitamin and mineral premix provided the following amounts per kilogram of premix: vitamin A, ≥450,000 IU/kg; vitamin D3, 300,000–400,000 IU/kg; vitamin E, ≥8000 IU/kg; vitamin K3, ≥800 mg/kg; vitamin B1, ≥1000 mg/kg; vitamin B2, ≥1000 mg/kg; vitamin B6, ≥1000 mg/kg; D-pantothenic acid, ≥3500 mg/kg; folic acid, ≥500 mg/kg; D-biotin, ≥5 mg/kg; vitamin C (L-ascorbyl-2-phosphate), ≥10,500 mg/kg; inositol, ≥12,000 mg/kg; copper, 200–500 mg/kg; iron, 20,000–30,000 mg/kg; manganese, 2000–6000 mg/kg; zinc, 6000–13,500 mg/kg; magnesium, 12,000–20,000 mg/kg. ^b^ Gross energy was calculated using energy equivalents 23.64, 39.54, and 17.15 kJ g^−1^ for protein, lipid, and digestible carbohydrate, respectively. ^c^ The soybean meal was a commercially produced, solvent-extracted, and heat-treated product. No additional heat treatment was applied before diet preparation.

**Table 2:** Primer pair sequences used in real-time PCR.

Target Gene	Primer Sequence (5′-3′)	Tm (°C)	Efficiency (%)
BAG	F: TCGCAGAAGAGCAGAAGGT	60.3	96
	R: CTATCAAGCAGGCTGTCAAAC	60.1	
BCL-2	F: GAGCAACAGTTGCCATCCACGAC	60.8	98
	R: CCTTCTCCACGCACTCCACACAC	61.2	
Caspase 3	F: GCTGGAGAACATCTTGGTTGTC	59.2	95
	R: TCTTGTAGCAGTAAGGGTCGGA	59.7	
Caspase 8	F: CTGTTTTGTTCTCTGACCTTGTG	61.5	99
	R: TCTTGTAGCAGTAAGGGTCGGA	60.9	
Caspase 10	F: CGACACATCTTTTACACCAC	58.6	94
	R: CCATACAGGACAGGATTACC	59.4	
IL-6	F: CTTCCCTGCGTGTGTCTCATTGG	61.0	97
	R: TGGGTTTGTTGTCCTCCCTCCTC	60.5	
IL-8	F: GTGACACGCTGGGATTTGAT	60.4	96
	R: GCCTGATGGTCCTCCTGAAG	60.7	
IL-1β	F: GCGACCGCAGTAAGAAAGAC	61.8	100
	R: TTGCTCCAGTTTCAGCACAG	61.4	
IL-10	F: AGCCAGCAGCATCATTACC	60.2	97
	R: GAACCAGGACGGACAGGAG	61.1	
Nrf2	F: AAGGAGGGGGAAGAACAAGG	62.3	96
	R: GAATAGAGGTCTGCAAGGCG	61.9	
Keap1	F: CCTCCACAAACCCACCAAA	59.5	95
	R: GCGTAGAAAAGCCCACTGA	60.0	
TGF-β	F: GGGTTTCCAACTTCGGCTATTC	62.1	98
	R: GGTCGGCTTATGTATCCACTTCC	61.7	
TNF-α	F: GTCCTGCTGTTTGCTTGGTGCT	60.7	98
	R: GAACGATGCCTGGCTGTAGACG	61.3	
Occludin	F: TCCTGGTTGTCATCGCCTTGGGT	61.4	99
	R: GACGGTCGGCTGGTGGTCCTCTC	62.0	
ZO-1	F: GCCCCCTCACCGTCCCCT	60.6	96
	R:CGGCTGCCCTGTTCCCAG	60.2	
Claudin 4	F: ACCATCATAATCTGCCTTCTCCC	60.1	95
	R: TCTTTTGCCTTTCATCCTCCACT	60.4	
Claudin 5	F: ATGAGGCGGTGAAGGCTAGAGTG	62.5	94
	R: GTGGGGGCGTATTTGATGGGGTA	62.2	
Claudin 1	F: CTTTTATCCGTCCATCGTCC	60.3	97
	R: ACATCACAGAGTCATCCAGC	60.8	
β-actin	F: GGACACGGAAAGGATTGACAG	61.6	99
	R: CGGAGTCTCGTTCGTTATCGG	61.2	
LMBV-MCP	F: CTCGCCACTTATGACAGCCTTGAC	60.9	98
	R: AACCCACGGGATAATGCTCTTTGAC	61.4	

F: forward primer; R: reverse primer.

**Table 3:** Effect of dietary supplementation of *Eucommia ulmoides* extract on the growth performance of largemouth bass.

	Groups						*p*-Value
Items	ele0	ele30	ele60	ele90	ele120	ele150	
FAW(g)	78.4 ± 3.25	81.1 ± 1.81	85.6 ± 4.75	83.0 ± 6.05	81.9 ± 1.35	81.9 ± 1.68	0.378
SR (%)	100.00	100.00	100.00	100.00	100.00	100.00	–
SGR(%/d)	3.23 ± 0.06 ^a^	3.33 ± 0.06 ^b^	3.37 ± 0.06 ^b^	3.33 ± 0.12 ^b^	3.33 ± 0.16 ^b^	3.16 ± 0.16 ^a^	0.023
FCR	0.960 ± 0.06	1.06 ± 0.03	1.01 ± 0.05	1.03 ± 0.08	1.04 ± 0.03	1.01 ± 0.05	0.612
WGR (%)	508 ± 19.45 ^a^	532 ± 18.15 ^ab^	561 ± 32.19 ^b^	555. ± 47.19 ^b^	543 ± 17.08 ^ab^	526 ± 16.35 ^a^	0.041

FAW, final average weight; SR, survival rate; SGR, specific growth rate; FCR, feed conversion rate; WGR, weight gain rate. Values are means ± SD (n = 3). Different superscript letters in the same row indicate significant differences among groups (*p* < 0.05, Tukey’s HSD). Exact *p*-values (ANOVA) are shown in the last column. n = 3 means 3 replicate nets per treatment.

**Table 4:** Effect of dietary supplementation with *Eucommia ulmoides* extract on the alpha diversity index of the intestinal flora of largemouth bass.

Items	ele0	ele60	ele120	*p*-Value
Shannon	1.786 ± 0.219 ^a^	1.576 ± 0.336 ^a^	1.549 ± 0.053 ^b^	0.042
Simpson	0.323 ± 0.048	0.319 ± 0.015	0.320 ± 0.026	0.893
OTUs	64.33 ± 7.02	55.33 ± 22.50	65.67 ± 19.14	0.761

Values are means ± SD (n = 3). Values labeled with different letters in the same row are significantly different (*p* < 0.05).

## Data Availability

The original contributions presented in this study are included in the article. Further inquiries can be directed to the corresponding authors.

## Abbreviations

ACP	acid phosphatase
ALT	alanine aminotransferase
AST	aspartate aminotransferase
BAG	Bcl-2-associated athanogene
BCL-2	B-cell lymphoma 2
CAT	catalase
C3	complement 3
C4	complement 4
Caspase	cysteinyl aspartate-specific protease
FCR	feed conversion ratio
GSH	glutathione
GSH-PX	glutathione peroxidase
HDL	high-density lipoprotein
IgM	immunoglobulin M
IL-1β	interleukin 1β
IL-8	interleukin 8
IL-10	interleukin 10
Keap1	Kelch-like ECH-associated protein 1
LDL	low-density lipoprotein
LPS	lipopolysaccharide
MDA	malondialdehyde
Nrf2	nuclear factor erythroid 2-related factor 2
qPCR	quantitative polymerase chain reaction
SGR	specific growth rate
SOD	superoxide dismutase
T-AOC	total antioxidant capacity
TC	total cholesterol
TGF-β1	transforming growth factor β1
TG	triglyceride
TNF-α	tumor necrosis factor α
WGR	weight gain rate
ZO-1	zonula occludens 1
LMBV-MCP	largemouth bass virus major capsid protein
